# Reply from authors: Formation of colloid film in open-reservoir cardiopulmonary bypass

**DOI:** 10.1016/j.xjon.2023.02.009

**Published:** 2023-02-24

**Authors:** Steven Toh, Mohamed Zeinah, Amer Harky

**Affiliations:** a Translational and Clinical Research Institute, Newcastle University, Newcastle upon Tyne, United Kingdom; b Faculty of Medicine, Ain Sham University, Cairo, Egypt; c Department of Cardiothoracic Surgery, Liverpool Heart and Chest Hospital, Liverpool, United Kingdom

Reply to the Editor:

**Figure:**
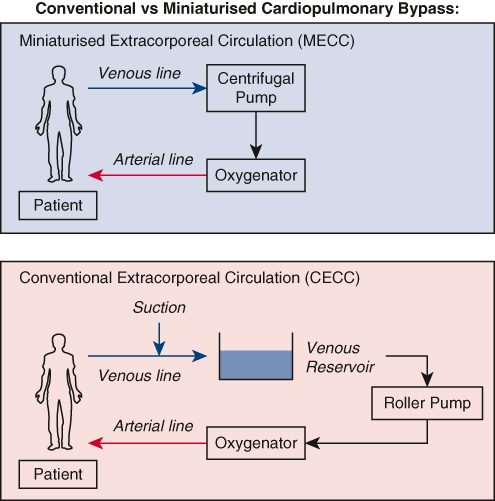

The authors reported no conflicts of interest.

The *Journal* policy requires editors and reviewers to disclose conflicts of interest and to decline handling or reviewing manuscripts for which they may have a conflict of interest. The editors and reviewers of this article have no conflicts of interest.

We thank the author for his interest in our article titled “Conventional Versus Miniaturized Cardiopulmonary Bypass: A Systematic Review and Meta-Analysis.”^1^

It is well known that cardiopulmonary bypass induces a systemic inflammatory response due to the contact of the patient's blood with nonendothelial surfaces and air, which is accompanied by an increase in cytokine levels. The attenuation of this inflammatory response is associated with a reduction of morbidity and mortality after cardiac surgery.

We note that the author reported the formation of a colloid film as an incidental finding during the administration of polygeline (Emagel) for volume integration in the establishment of cardiopulmonary bypass. The use of a colloid film as a solution to temporarily reduce air–blood contact in optimized open circuits has both potential benefits and risks.

**Figure 1:**
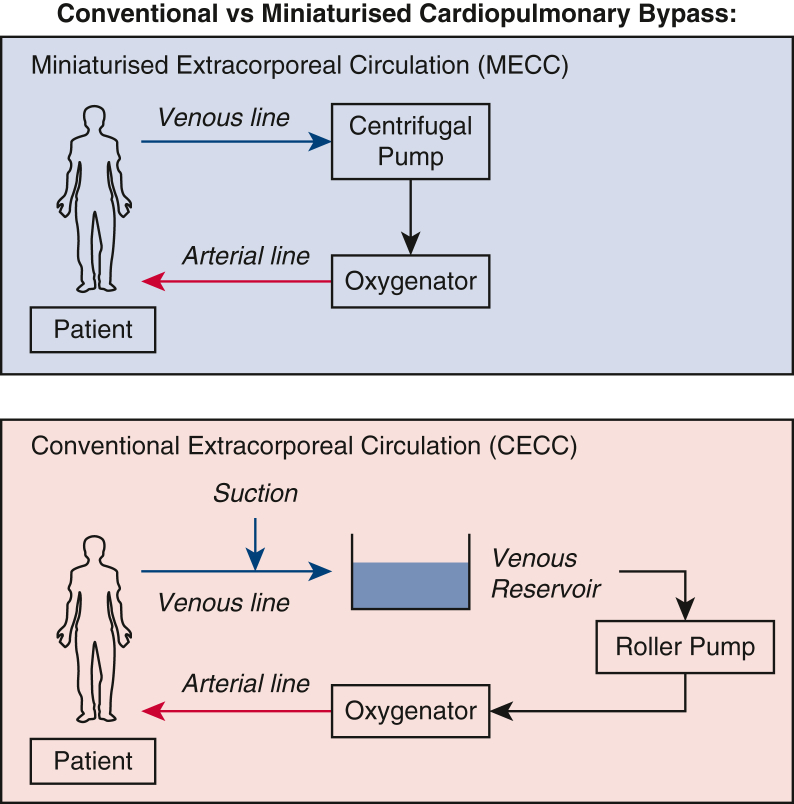
Diagram comparing process of conventional cardiopulmonary bypass (*bottom*) and miniaturized cardiopulmonary bypass (*top*).

The elimination of the air–blood interface reduces contact activation of blood components when exposed to air, especially with longer perfusion times (Figure 1). However, the clinical benefits of this are controversial and not well proven.^2^^,^^3^

Colloid solutions are effective in increasing circulatory blood volume and maintaining colloid osmotic pressure in cardiopulmonary bypass. However, the use of such solutions increases the cost of cardiac surgery and may cause adverse reactions such as histamine release, resulting in hypotension, bronchospasm, and skin rash.^4^^,^^5^

The hemodilution effect caused by large amounts of colloids could result in an increased risk of stroke or other neurological events through cerebral hypoperfusion.^6^, ^7^ A stagnant colloid film also may be a point of stasis that increases the risk of clinical coagulopathy.

Although the formation of a colloid film reduces air–blood contact for a short period of time, the clinical benefits of this are not well investigated. The use of solutions to limit air–blood contact in optimized open circuits is thought provoking, and more research is needed to fully evaluate its effectiveness and safety.
